# Improving exposure for transoral oropharyngeal surgery with the floor of mouth window: a cadaveric feasibility study

**DOI:** 10.1186/s40463-019-0383-2

**Published:** 2019-11-12

**Authors:** Jeffson Chung, Adam Bender-Heine, H. Wayne Lambert

**Affiliations:** 1 Department of Otolaryngology – Head & Neck Surgery, West Virginia University, 1 Medical Center Drive, Health Sciences South, Rm 4532, Morgantown, WV 26506 USA; 2 Department of Otolaryngology – Head & Neck Surgery, West Virginia University, 1 Medical Center Drive, Health Sciences South, Rm 4500, Morgantown, WV 26506 USA; 3 Department of Pathology, Anatomy and Laboratory Medicine, West Virginia University, 1 Medical Center Drive, Health Sciences North, Rm 4054, Morgantown, WV 26506 USA

**Keywords:** Transoral robotic surgery, Transoral laser microsurgery, Oropharyngeal carcinoma

## Abstract

**Background:**

Transoral robotic and laser surgery is rising in popularity due to the increasing incidence of Human Papilloma Virus (HPV) related oropharyngeal cancer. However, adequate exposure of the tongue base remains a major hurdle in many cases. This study introduces a novel surgical technique called the Floor of Mouth Window, which can be used to improve tongue base exposure at the time of transoral surgery.

**Methods:**

This is a preclinical anatomic cadaver study. Seven fresh cadavers were used for this study. Exposure of the tongue base was compared between conventional mouth gags – the Feyh-Kastenbauer and McIvor – and our novel procedure, the Floor of Mouth Window. Exposure was compared subjectively using endoscopic and extracorporeal photographs, as well as objective measurements of inter-incisor distance, and oral cavity volume.

**Results:**

The exposure achieved by the Floor of Mouth Window technique was superior to the mouth gags. Inter-incisor distance and oral cavity volume measurements were all more favorable with the Floor of Mouth Window. This technique allowed for successful transoral laser tongue base and tonsil resection without the use of gags or scopes.

**Conclusion:**

The Floor of Mouth Window is an adjunctive procedure that simply and reliably improved exposure for transoral oropharyngeal surgery in this cadaveric feasibility study. This improved exposure may help increase the adoption of transoral surgery and reduce the number of aborted cases due to anatomic limitations.

## Background

Transoral surgery is gaining popularity as a treatment option for oropharyngeal cancers [1], which is primarily due to the increasing incidence of small primaries often seen with human papilloma virus (HPV) related disease, as well as the advent of technologies allowing for minimally invasive surgery. Studies demonstrating the efficacy of surgery in affecting locoregional control, disease specific survival, and overall survival, have further popularized its use [2, 3]. Moreover, surgery fits well within the modern treatment de-intensification paradigm by clearing observable disease and reducing or even eliminating adjuvant radiation, thereby decreasing long-term radiation-induced side effects.

Transoral surgery on the oropharynx is currently performed with the assistance of robots, fiberoptic lasers and microscopes, or with endoscopes and conventional monopolar cautery. Regardless of technique, a good view of the oropharynx with sufficient access for instruments is the most crucial part of the surgery and can present a major challenge in some cases. If an adequate view and sufficient working space for instruments are not achievable, the patient may not be a candidate for transoral surgery and all its attendant benefits. Studies have shown that as many as 7.8–18% of patients have been deemed unsuitable surgical candidates due to these limitations, and these numbers do not include patients who went on to receive transoral surgery despite suboptimal exposure and significant struggle on the part of the surgeon [4, 5].

This cadaveric feasibility study describes and examines a novel surgical procedure called the “Floor of Mouth Window” that can be performed at the time of transoral surgery to improve operative exposure simply and reliably, while eliminating the need for mouth gags.

## Methods

This study was reviewed and approved by the West Virginia State Anatomical Board and the West Virginia University Institutional Review Board (protocol #1711850572). A total of seven fresh cadavers were donated by the West Virginia University Human Gift Registry. No demographic or medical information was obtained about any of the cadavers. No anatomic abnormalities or prior surgeries were evident in the head and neck of any of the specimens.

For each cadaver, we compared the optimal exposure achievable for transoral oropharyngeal surgery using the Feyh-Kastenbauer (FK) and McIvor mouth gags to that achievable using the Floor of Mouth Window technique (detailed below). For the mouth gags, we used all the available tongue blades and selected the ones that best exposed the base of tongue. A plastic lip and cheek retractor was used in all cases.

### Operative technique – floor of mouth window

**Fig. 1:**
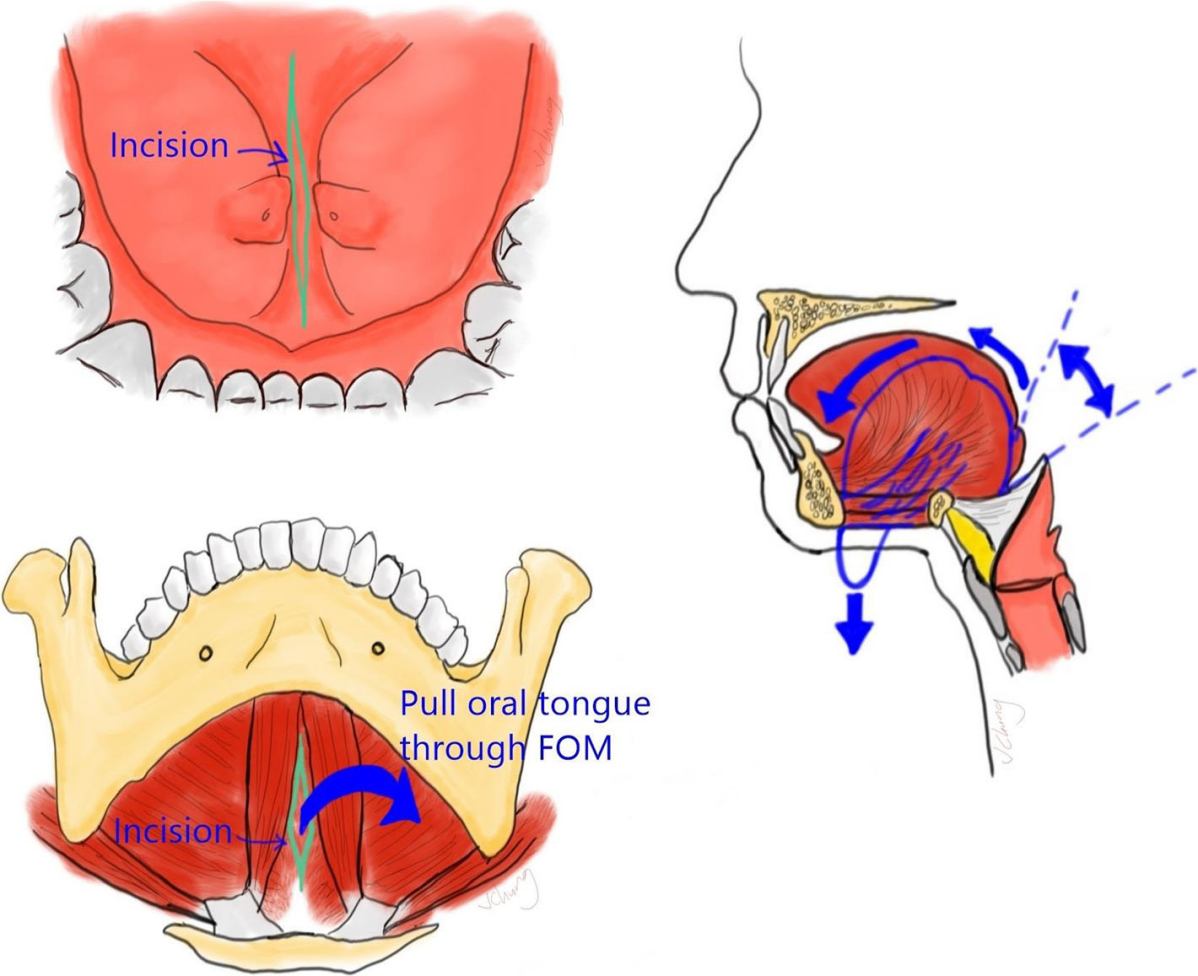
Illustration of how to perform the Floor of Mouth Window

**Fig. 2:**
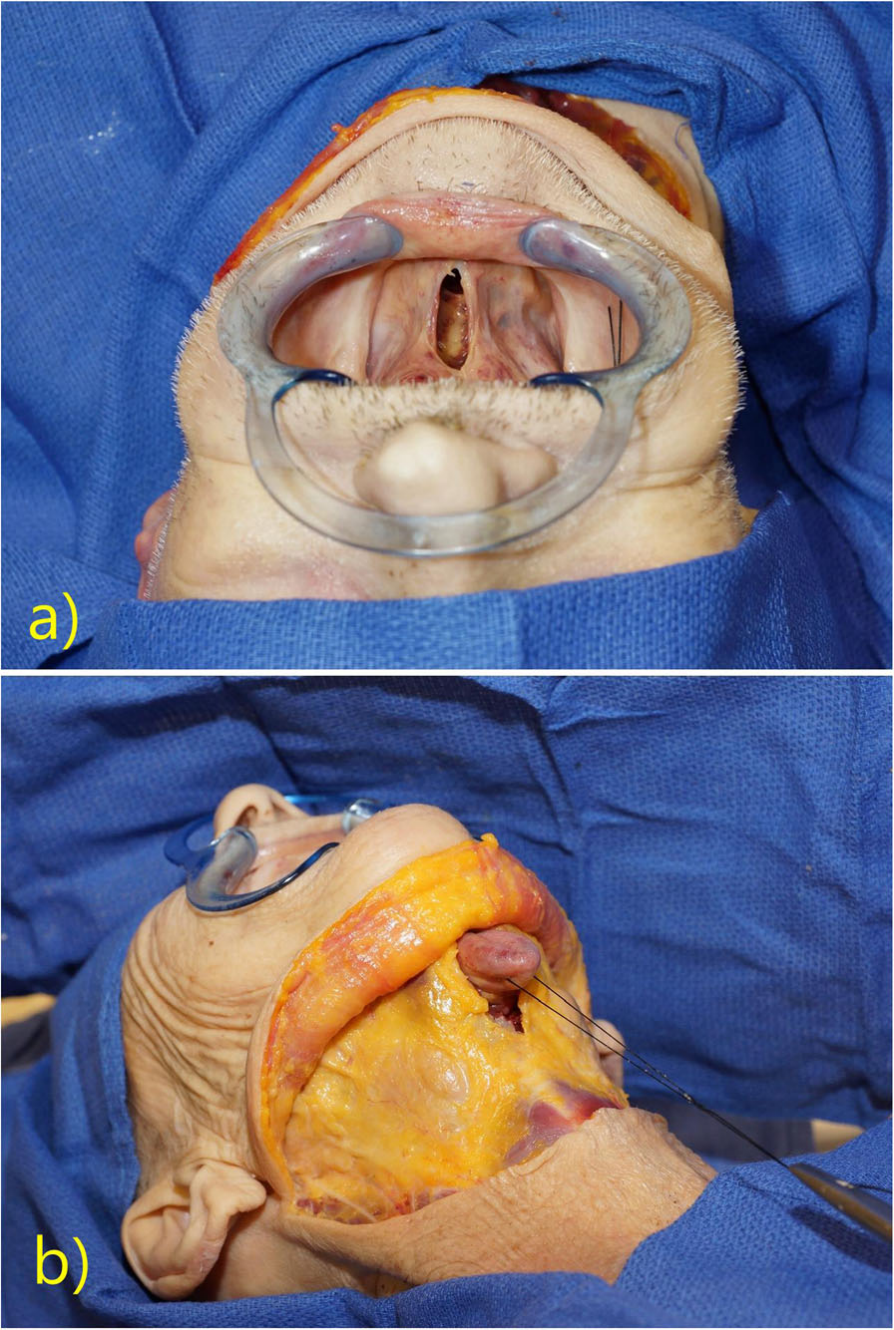
**a** The Floor of Mouth Window and **b** traction on the oral tongue within the submental space

First, an apron incision was made as per routine for bilateral neck dissections. The subplatysmal flap was then elevated superiorly to expose the submental triangle. Next, an intraoral incision was made in the midline floor of mouth between the sublingual caruncles, in the sagittal plane. Blunt dissection was then carried through this incision towards the submental space, between the genioglossus and geniohyoid muscle bellies, through the mylohyoid muscle, and between the anterior digastric muscle bellies. The Floor of Mouth Window thus communicated the anterior oral cavity with the submental space. A silk retention suture was then placed through the tip of the tongue and passed through the Floor of Mouth Window, thus aiding in the extrication of the oral tongue through the Floor of Mouth Window into the submental space. Traction on the oral tongue in the submental space without the use of mouth gags constituted the exposure achievable by the Floor of Mouth Window (Fig. 1 and Fig. 2**)**

### Comparison of exposures

For all seven of the cadavers, subjective comparison of the exposures offered by the mouth gag and the Floor of Mouth Window were done using extracorporeal and intra-oral endoscopic photographs. Both 0 and 30 degree endoscopes were used. After successfully testing feasibility in the first three cadavers, we attempted quantitative comparisons, which consisted of measurements of inter-incisor distance in four cadavers and volume of the intraoral working space in three. The measurement of inter-incisor distance was measured as the available working space between the upper and lower incisors or alveoli depending on the presence of teeth. When a mouth gag was used, the available working distance was similarly measured but between the tongue blade, which rested over the lower incisors, and the frame, which hooked over the upper incisors. The volume measurement was performed by clamping off the trachea and esophagus and filling the oral cavity with known volumes of water until the water level reached the anterior-most part of the upper alveolus. Due to the small sample size, tests of significance such as the Wilcoxon signed rank test for paired samples and the Mann-Whitney U test could not be applied.

Finally, as a test of the adequacy of exposure, a transoral laser tongue base and palatine tonsil resection was attempted in one of the cadavers using the Floor of Mouth Window technique. The OmniGuide Intelliguide CO2 laser system (FELS-25A) with the Elevate ENT handpiece and Elevate Elite ENT fiber was used (OmniGuide, Inc., Cambridge, MA).

## Results

**Fig. 3:**
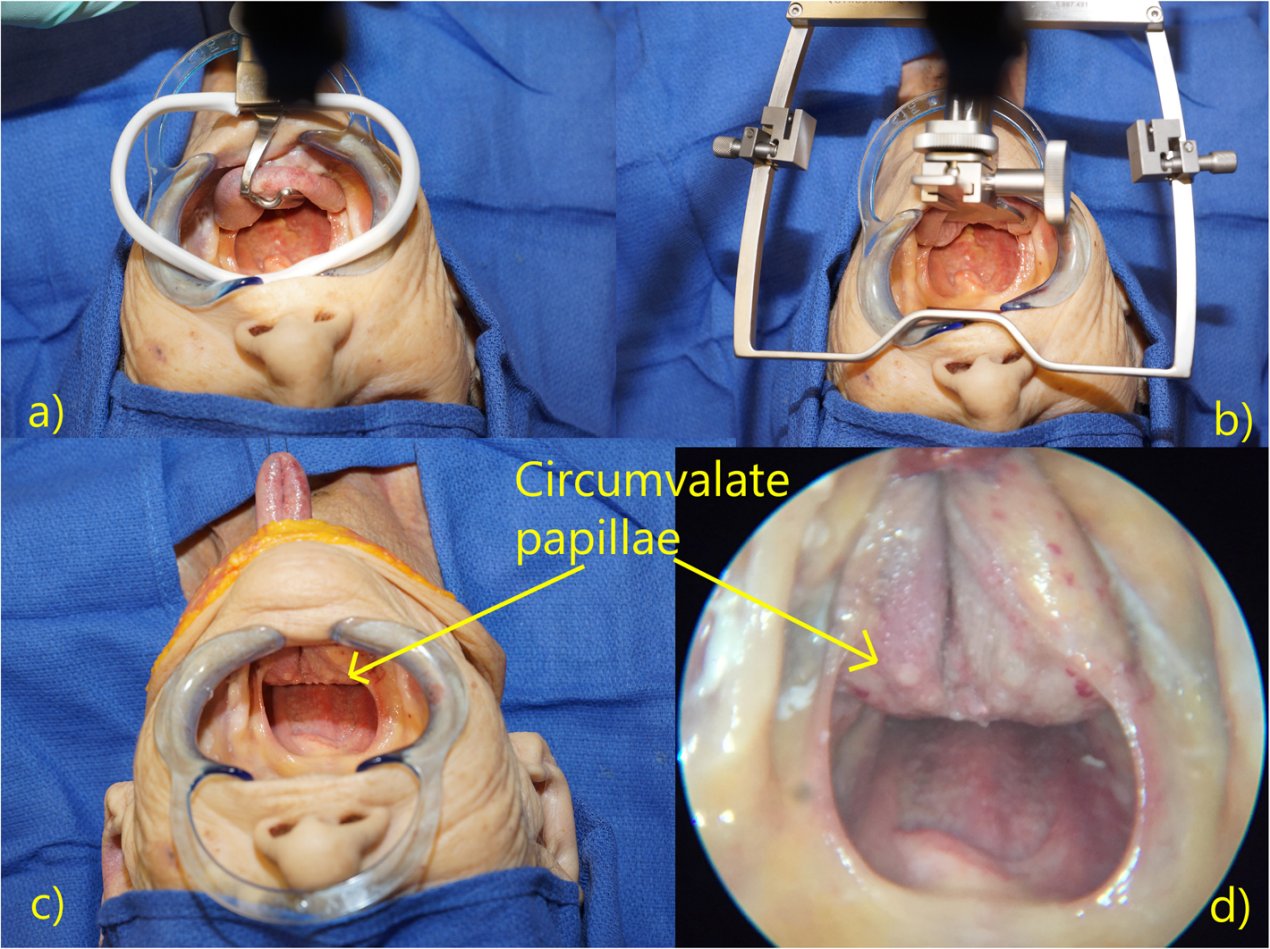
Exposures offered by the **a**) McIvor gag, **b**) Feyh-Kastenbauer gag, and **c**) Floor of Mouth Window. Note that the circumvallate papillae is easily visible transorally without the aid of endoscopes. **d**) Endoscopic view with the Floor of Mouth Window

In all cadaveric specimens, the exposure offered by the novel Floor of Mouth Window technique was subjectively at least as good if not better than that achieved using the mouth gags. In most cases, the circumvallate papillae were made visible transorally even without the aid of endoscopes (Fig. 3). In all four cases where inter-incisor distances were measured, the Floor of Mouth Window offered the largest measurement, averaging an improvement of 5 mm over the McIvor mouth gag (Table 1). Similarly, in all three cases where oral cavity volume measurements were taken, the Floor of Mouth Window procedure created the largest working space, averaging 52 mL more than the McIvor gag (Table 2).

**Table 1:** Inter-incisor distance achieved using the McIvor gag versus Floor of Mouth Window (FOMW)

		Inter-incisor distance (mm)		
Cadaver	Dental status	McIvor gag	FOMW	Difference
1	Edentulous	30	40	+ 10
2	All incisors present	25	30	+ 5
3	Edentulous	38	42	+ 4
4	Upper incisors absent	32	34	+ 2

**Table 2:** Oral cavity volume when using the McIvor gag versus Floor of Mouth Window (FOMW)

	Oral cavity volume (mL)		
Cadaver	McIvor gag	FOMW	Difference
1	185	240	+ 55
2	210	270	+ 60
3	230	270	+ 40

**Fig. 4:**
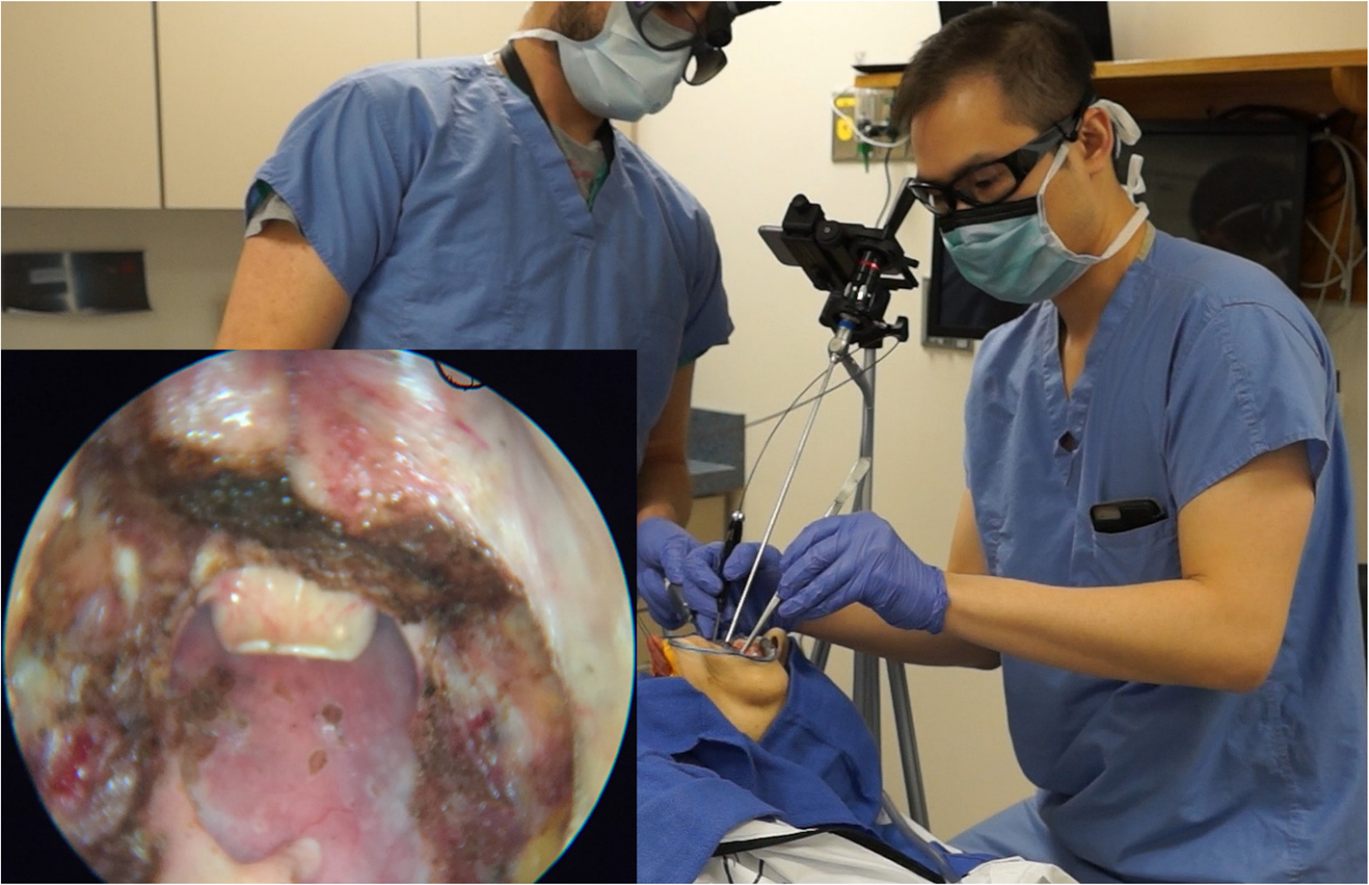
Transoral laser resection of the tongue base and tonsils without the aid of mouth gags or endoscopes (except to take the photograph shown in the inset)


Finally, we demonstrated the adequacy of the Floor of Mouth Window exposure by successfully performing a transoral laser resection of the tongue base and palatine tonsils in one of the cadavers without the aid of mouth gags or even endoscopes (Fig. 4 and Additional file 1: Video S1).

**Additional file 1: Video S1.** Sped-up video demonstrating transoral laser tongue base excision using the Floor of Mouth Window exposure in a cadaver. Note that this was performed without the use endoscopic assistance, except to film the procedure.


## Discussion

Regardless of whether transoral surgery is performed by robots or laser, exposure and access have thus far relied on mouth gags. While mouth gags have allowed for successful transoral surgery in the majority of attempts in expert hands, gags are far from a perfect solution. This is evident from the many attempts to improve surgical access to the oropharynx throughout history [6]. Examples include the transhyoid and suprahyoid pharyngotomies, lingual release pull-through, and even lip-sparing mandibulotomy approaches [7–13]. Others have sought to solve the access problem by innovating on the gag itself. Transoral surgeons have used the McIvor, Boyle-Davis, Dingman, FK, and LARS retractors, including unnamed variants, plus the myriad of tongue blades that accompany all this hardware [14–17]. Some opt for laryngoscopes, and these too come in many shapes and sizes. With so many different gags and laryngoscopes available, there is clearly no ideal solution among them.

This fact is further evidenced by ongoing efforts to improve access even now amongst experienced transoral surgeons. Miller (2016) published a small series using only a retention stitch on the tongue without a gag [18]. However, this approach drapes the tongue over the lower incisors which significantly reduces the inter-incisor working distance. Moore et al. (2017) published a small series where cadavers and patients were put into a seated position for transoral robotic surgery (TORS) [19]. However, this technique required not only a special OR table, but it also required the robot be docked at an unfamiliar angle. The patient-side surgeon also has poor access to the site, requiring the robot to be withdrawn and the patient leveled in the case of accidental extubation or uncontrollable bleeding. Moreover, the oropharyngeal access demonstrated by intraoperative photos appeared marginally better than conventional positioning.

The Floor of Mouth Window technique improves access by addressing two key limitations to transoral surgery not addressed by other surgical approaches. The first limitation is that the oral cavity is a fixed volume box. Mouth gags and laryngoscopes work by retracting the oral tongue but because the tongue is non-compressible, there is no net gain in working space; it simply deforms under the tongue blade. Moreover, the bulky retraction system itself occupies precious volume within the oral cavity. The tongue deformation resulting from the use of gags alters the anatomy and can hinder the surgeon’s assessment of the tumor margins, often requiring trials of various tongue blades and adjustments – sometimes mid-resection – to arrive at an acceptable exposure. The metal tongue blade itself obscures most of the operative field by covering up the tongue. Pressure from the tongue blade has also been known to cause complications such as neuropraxias and ventral tongue lacerations from compression against the lower incisors [20]. The Floor of Mouth Window on the other hand, addresses the space limitation by extricating the oral tongue from the oral cavity and eliminating retractors altogether, thereby maximizing the space available for endoscopes or other instruments. We also found that the Floor of Mouth Window improved the inter-incisor distance, not necessarily from increased mouth opening, but because eliminating the retractor also eliminated a tongue blade that is placed over the lower incisors and a frame that hooks over the upper incisors, both of which reduce the available working distance between the upper and lower incisors.

The second limitation of transoral surgery is the often tangential view of the tongue base that occurs with the use of conventional gags. The mouth gag compresses the tongue in a purely caudal direction, which does not greatly improve line of sight. While this limitation is somewhat mitigated by using angled endoscopes, this requires additional expensive instrumentation; it is also not an option for transoral laser microsurgery (TLM). The Floor of Mouth Window addresses this issue by rotating the base of tongue towards the oral cavity, allowing for a more en face view of the tongue base (Fig. 1); this direct line of sight of the tongue base was what allowed us to perform a laser tongue base resection without the aid of endoscopes or microscopes.

Note that the Floor of Mouth Window technique is different from the previously described pharyngotomy approaches in two ways. The first difference is that our technique does not require a communication between the primary resection site and the neck as it uses an incision in the oral cavity, not oropharynx. The second is that pharyngotomy approaches involve the delivery of the tongue base into the neck for a traditional open resection, whereas the Floor of Mouth Window is used with natural orifice transoral techniques that take advantage of the superb visualization offered by stereoscopic endoscopes or microscopes [21].

**Fig. 5:**
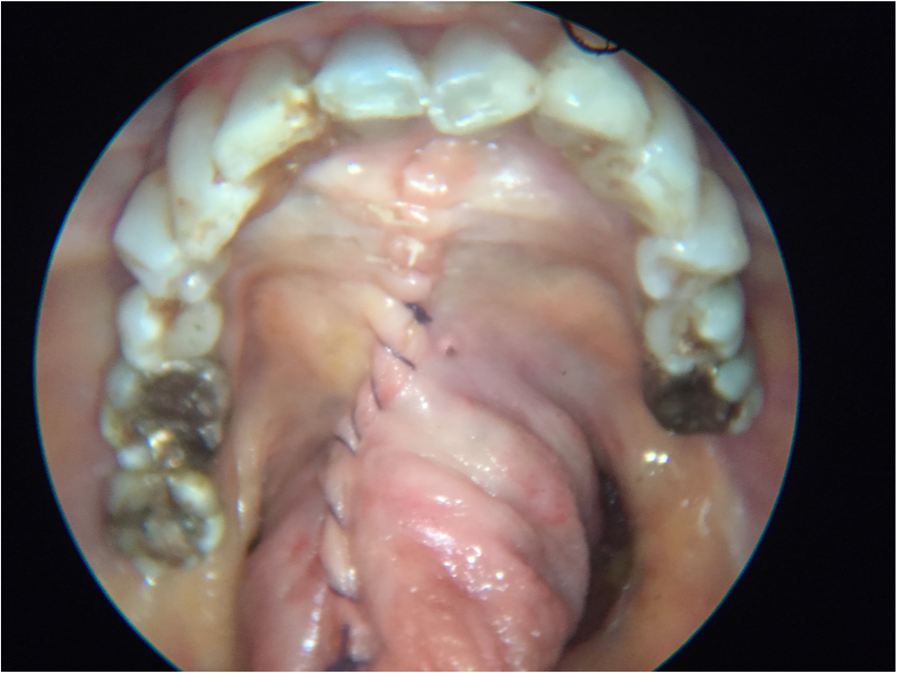
Primary closure of the Floor of Mouth Window in layers

The Floor of Mouth Window approach is not without its shortcomings. First, it requires a concomitant neck dissection which may or may not be every surgeon’s practice; some prefer to stage the surgeries. Also, by virtue of requiring a neck dissection incision, this procedure would be applicable only to malignant disease and not to benign indications for TORS or TLM. An apron style neck incision is also preferred, which may not be every surgeon’s practice. The Floor of Mouth Window does require an additional floor of mouth incision, which carries its own risks, including possible submandibular duct injury or obstruction. However, the dissection is performed between the sublingual caruncles through a relatively avascular midline raphe, far from both the lingual and hypoglossal nerves, and as a result, morbidities are likely to be minimal. There is a theoretical risk of fistula, but it is likely small given that far larger defects at the primary oropharyngeal resection sites have been shown to have low fistula rates [22]. Furthermore, the Floor of Mouth Window can be primarily closed in layers underneath intact submental skin, minimizing the chance of fistula (Fig. 5). As for the risk of tumor seeding, this procedure involves extricating the oral tongue through the submental space, neither of which are typically involved in an oropharyngeal cancer [23]. A level 1a nodal dissection, however, can be performed as per the surgeon’s preference. As a result, we see no reason that the Floor of Mouth Window should prove to be less safe and effective than conventional exposure techniques, and we see no risks greater than what is currently in practice.

The limitations of this study include the fact that this technique was trialed on a small number of cadavers, which represented a limited number of anatomic variants. These cadavers also did not have oropharyngeal tumors which may introduce challenges to exposure not present in normal anatomy. And because our study was performed on cadavers, we are unable to comment on the risk of adverse events such as airway swelling requiring tracheotomy, or nerve traction or compression injuries. Finally, our evaluation of exposure was largely subjective, though attempts were made to quantify the improvement in exposure using inter-incisor distance and volume of the intraoral working space. Still, we felt that there was an appreciable gain in visualization and working space using the Floor of Mouth Window technique as demonstrated by the photos. Future directions will be to expand the number of cadavers, use the Friedman classification as a method of assessment, and to trial this procedure in actual patients.

Transoral surgery for oropharyngeal cancer has increased in popularity due to its myriad of benefits. However, one of the challenges limiting its use to specially trained and experienced transoral surgeons is the difficulty in obtaining the required exposure in some patients. We hope that the Floor of Mouth Window technique will make it consistently easier to proceed with transoral surgery in these patients and minimize the need to abort. It may even potentially improve access for free flap reconstruction following transoral surgery [24]. It is a simple procedure to perform that does not require any additional training or special equipment, which can lower the barrier to entry for transoral surgery and increase widespread adoption. Moreover, this procedure addresses the limitations associated with mouth gags, and as a result, is applicable to both robot and laser technologies, as well as future innovations, including single port or flexible robot systems [25].

## Conclusions

The Floor of Mouth Window is an adjunctive operative procedure that can be performed at the time of transoral surgery and neck dissection for oropharyngeal cancer. It improves exposure in this cadaveric model in a simple and consistent way and does so by addressing the limitations associated with mouth gags. We have shown that it is possible to perform tongue base resection without a mouth gag system using this procedure. The hope is that the Floor of Mouth Window will overcome anatomic challenges and render more patients candidates for transoral surgery.

## Data Availability

The datasets used and/or analysed during the current study are available from the corresponding author on reasonable request.

## Abbreviations

FK: Feyh-Kastenbauer
HPV: Human Papilloma Virus
TLM: Transoral laser microsurgery
TORS: Transoral robotic surgery
